# Inverted organic solar cells with non-clustering bathocuproine (BCP) cathode interlayers obtained by fullerene doping

**DOI:** 10.1038/s41598-019-46854-w

**Published:** 2019-07-18

**Authors:** Fatemeh Jafari, Bhushan R. Patil, Fatemeh Mohtaram, André L. Fernandes Cauduro, Horst-Günter Rubahn, Abbas Behjat, Morten Madsen

**Affiliations:** 1 SDU NanoSYD, Mads Clausen Institute, University of Southern Denmark, Alsion 2, 6400 Sønderborg, Denmark; 2 Atomic and Molecular Groups, Faculty of Physics, Yazd University, Yazd, Iran; 3 Department of Textile Engineering, Isfahan University of Technology, Isfahan, 84156-83111 Iran; 4 National Center for Electron Microscopy, Molecular Foundry, Lawrence Berkeley National Laboratory, One Cyclotron Road, 94720 Berkeley, California United States

**Keywords:** Devices for energy harvesting, Electronic devices

## Abstract

Bathocuproine (BCP) is a well-studied cathode interlayer in organic photovoltaic (OPV) devices, where it for standard device configurations has demonstrated improved electron extraction as well as exciton blocking properties, leading to high device efficiencies. For inverted devices, however, BCP interlayers has shown to lead to device failure, mainly due to the clustering of BCP molecules on indium tin oxide (ITO) surfaces, which is a significant problem during scale-up of the OPV devices. In this work, we introduce C_70_ doped BCP thin films as cathode interlayers in inverted OPV devices. We demonstrate that the interlayer forms smooth films on ITO surfaces, resulting from the introduction of C_70_ molecules into the BCP film, and that these films possess both improved electron extraction as well exciton blocking properties, as evidenced by electron-only devices and photoluminescence studies, respectively. Importantly, the improved cathode interlayers leads to well-functioning large area (100 mm^2^) devices, showing a device yield of 100%. This is in strong contrast to inverted devices based on pure BCP layers. These results are founded by the effective suppression of BCP clustering from C_70_, along with the electron transport and exciton blocking properties of the two materials, which thus presents a route for its integration as an interlayer material towards up-scaled inverted OPV devices.

## Introduction

Organic photovoltaic (OPV) devices based on small molecules and polymers have attracted a great interest in recent decades due to their appealing properties being eco-friendly, potentially low-cost, lightweight, mechanically flexible and semi-transparent^1–4^. Within vacuum processed OPV, several research and development efforts have been conducted over the years, and the commercial potential of the technology is also demonstrated by e.g. the company Heliatek, showing that mass production of the technology from roll-to-roll technology is a viable route for commercialization^5–7^. Owing to the fast progressing research in the OPV field in general, the power conversion efficiency (PCE) of single-junction OPVs has today reached 14.9%^8,9^, while the PCE of tandem OPV cells has crossed over 17%^10^, employing typically used bulk-heterojunctions as the active layers. While bilayer devices have show less progress, they potentially offer a route for minimizing charge recombination effects due to the separated electron and hole charge transport pathways. L. Calio *et al*. recently reported the PCE of 5.66% for vacuum deposited bilayer OPVs based on tetraphenyldibenzoperiflanthen (DBP) donor and Fullerene (C_70_) acceptor incorporating efficient exciton blocking layers^11^.

Fullerenes and their derivatives are still widely used as electron acceptors in OPV devices due to their high electron mobility as well as electron affinity, which promotes efficient charge separation at the organic electron donor/acceptor (D/A) interface, when used in combination with a suitable donor such as DBP investigated in this work^12–16^. In order to efficiently extract electrons out from the electron acceptor, and minimize losses at the cathode interface, a layer that acts both as exciton blocking (EBL) and electron transporting (ETL) is needed in between the cathode and electron acceptor layer^17,18^. Such combined EBLs and ETLs blocks photo-generated excitons from quenching at the cathode electrode, while extracting electrons (and not holes) efficiently out from the acceptor, through an ideally zero energy barrier at that interface^19^. Potentially, such layers also provide a transparent spacer to optimize the optical field distribution within the active layer, and thus enhance the OPV performance even further^20–22^. Peumans *et al*.^23^ introduced for the first time a BCP (2,9-dimethyl-4,7-diphenyl-1,10-phenanthroline) layer as a combined EBL and ETL in between the electron acceptor and cathode in bilayer, standard configuration OPV cells based on CuPc and fullerene, C_60._

Bathocuproine (BCP) is widely used as ETL and EBL material in OPVs. BCP has a highest occupied molecular orbital (HOMO) level at 7.0 eV and lowest unoccupied molecular orbital (LUMO) at 3.5 eV^24,25^. Despite the relatively high lying LUMO level, BCP efficiently transports electrons from the acceptor to the cathode due to the formation of a BCP-metal complex, formed when the metal cathode is evaporated on top of the BCP layer^26^. Additionally, BCP works as an EBL and electron selective contact due to its relatively low lying HOMO level of 7.0 eV compared to the HOMO level of e.g. C_70_ at 6.1 eV^27^. The exciton blocking properties of BCP results in a higher charge generation yield at the D-A interface, which again leads to enhanced device performance through enhanced short-circuit current densities^28–30^. Work reported by Gommans *et al*. has documented that BCP also can act as an optical spacer layer, to best exploit optical interference effects in OPV cells^31^.

Several studies have focused on the function of BCP as ETL and EBL in OPV devices with standard device architecture^24,31,32^, highlighting the aforementioned properies. In our previous study, an area-dependent behavior of BCP used as ETL and EBL in inverted OPV devices was reported^33^. It was observed that while scaling up the OPV device area, the performance and device yield of the inverted OPV devices decrease significantly compared to standard configuration cells, which was demonstrated to be due to the clustering of BCP on ITO surfaces^33,34^. While BCP on small device areas works well as both EBL and ETL, the probability of BCP clusters penetrating the active layer (approx. 50 nm thick in that study) increases for increased device area. This potentially results in electrical shunting of the inverted OPV devices, which dramatically decrease the device yield for up-scaled cells. In recent work, this has also been demonstrated to lead to faster degradation of inverted OPV devices based on pure BCP ETL and EBL layers^35^. The integration of Ag doped BCP layers in inverted OPVs as buffer layers has previously been reported on^36,37^. However, although these layers provide improved electrical properties, Ag doped BCP may lead to unwanted exciton quenching processes in the fullerene acceptor layer and thus deteriorate the device performance^37^. Such quenching processes between metals and adsorbed molecules are well-know^38^.

Incorporation of interlayers or buffer layers fabricated from a blend of two or more organic materials is a common practice in OPV devices. The blended layers potentially improve the device performance by enhancing the electrical properties at the respective interface (interlayers), and/or the optical properties of the devices (interlayers or buffer layers)^17,39,40^. Bartynski *et al*. used a blend of C_60_ and BCP layer as ETL and EBL in standard OPV devices that improved the electron conductivity, while efficiently blocking excitons and reducing exciton-polaron recombination^27^. Furthermore, Xiao *et al*. reported that a blend of BPhen:C_60_ increases the electron conductivity and, as well, decreases exciton recombination effects in the devices^41^. Liu *et al*. used a BCP:C_60_ layer as EBL and ETL in standard configuration OPV devices to optimize the optical properties of the devices, and also the device lifetime^42^. However, compared to C_60,_ C_70_ offers higher stability upon air exposure^43^, and also a higher conductivity^43^, which may be beneficial when used as an interlayer material in organic photovoltaic devices.

**Figure 1:**
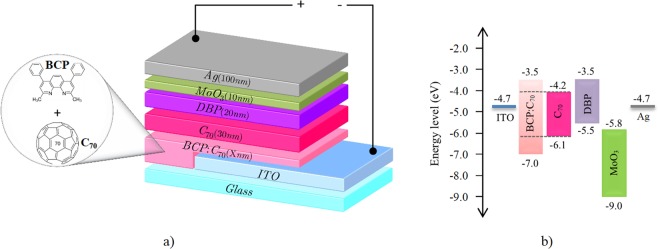
(**a**) Inverted OPV device architecture with BCP:C_70_ as ETL and EBL. (**b**) Energy level diagram of OPV with inverted configuration (the energy level of C_70_ in the BCP:C_70_ layer is indicated by the dashed lines).

In this work, we studied bathocuproine:fullerene (BCP:C_70_) acting as ELB and ETL functional blends in inverted architecture OPV devices, as sketched in Fig. 1a. The optimization of the BCP:C_70_ ratio as well as the thickness of the blend layer was investigated. The optimized BCP:C_70_ layers were employed in inverted OPV devices having active areas of up to 100 mm^2^, and the results were compared against inverted OPVs based on pure BCP layers. The investigation shows that the BCP:C_70_ blends suppress the clustering of BCP on top of ITO surfaces, leading to a significantly improved device performance and especially device yield for up-scaled inverted OPV devices.

## Results and Discussion

Figure 1 shows the inverted bilayer OPV device architecture studied in this work, having BCP:C_70_ as ETL and EBL, as well as a schematic energy diagram of the device stack made from literature reported energy level values. DBP possesses a high optical absorption strength in the visible wavelength regime and a HOMO level at 5.5 eV^44^, making it a good match to fullerene acceptors such as C_60_ and C_70_^11^.

**Figure 2:**
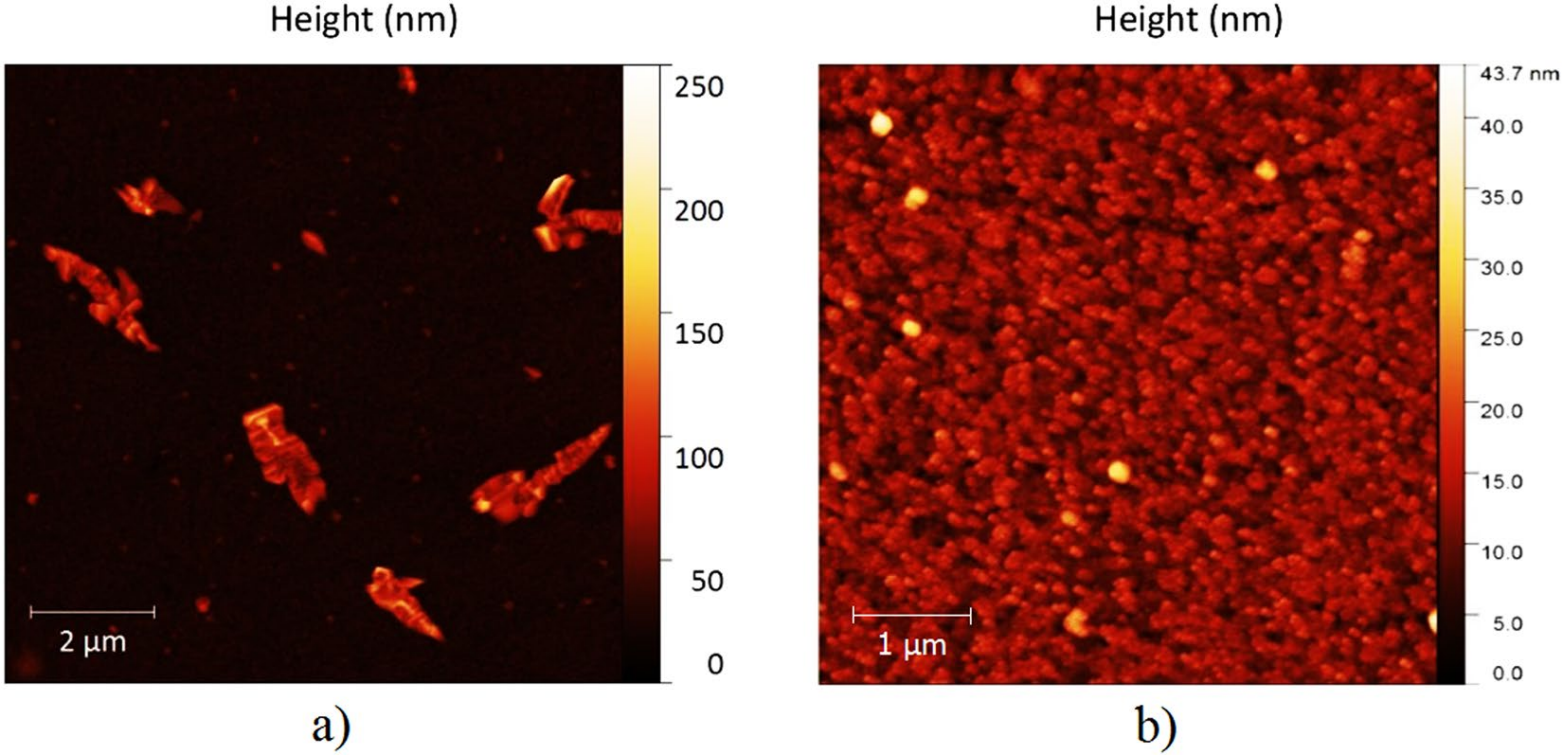
The AFM images comparing the morphology of 3 nm a) BCP and b) BCP:C_70_ (2:1) thin films on top of ITO/glass substrates.

Figure 2a shows an atomic force microscopy (AFM) image of 3 nm pure BCP deposited on top of an ITO coated glass substrate. The clustering of BCP occurs due to a large interface energy between ITO and BCP^33^, and may take place immediately after BCP deposition even at room temperature^34^. Such clustering can be explained by Ostwald ripening, in which some aggregates grow at the expense of others by adsorbing molecules from the surrounding surface area^45^. At larger surface area, the probability of forming clusters that cause device shunts are larger^33^, making device upscaling more challenging in inverted OPV architectures. One possible solution to overcome BCP aggregation is to conduct co-evaporation with another organic small molecule in order to obtain smoother films^34^. As shown in Fig. 2b, doping C_70_ molecules into the BCP film via co-evaporation is effective in preventing the aggregation of BCP molecules, resulting in a nano-grained surface on ITO.

As reported in our previous work, the optimized BCP ETL thickness for small area inverted DBP/C_70_ based bilayer OPV devices is 1.5 nm^33^, which was therefore chosen as initial ETL thickness in this work. Optimization results from 2 mm^2^ inverted OPV cells with various ratios of the BCP:C_70_ ETL and EBL are listed in Table S1, showing that devices with 2:1 ratio show slightly higher Fill Factor (FF) with an average value of 55%, V_oc_ with an average value of 0.82 V, as well as PCE reaching an average value of 2.28%. Even though the 2:1 blend films lead to reasonable device performance, an improvement in the short-circuit current density is not seen, compared to reference cells, which is otherwise expected from the exciton blocking properties of the interlayer. This could be due to the relatively low thickness (1.5 nm) of the blend layer.

**Figure 3:**
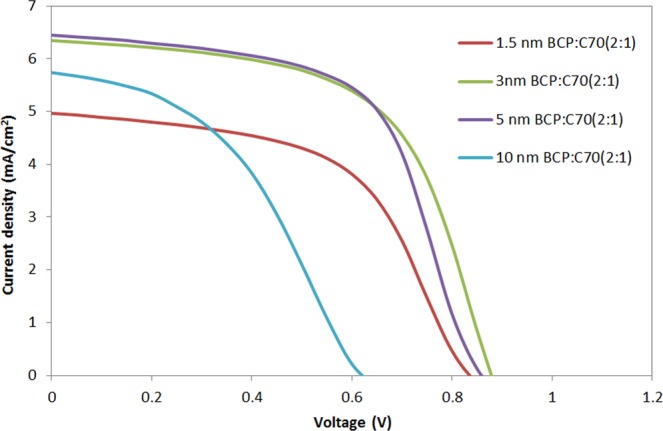
The JV characteristic of representative J-V curves for the inverted OPV devices with various thicknesses of the BCP:C_70_ (2:1) ETL and EBL.

**Figure 4:**
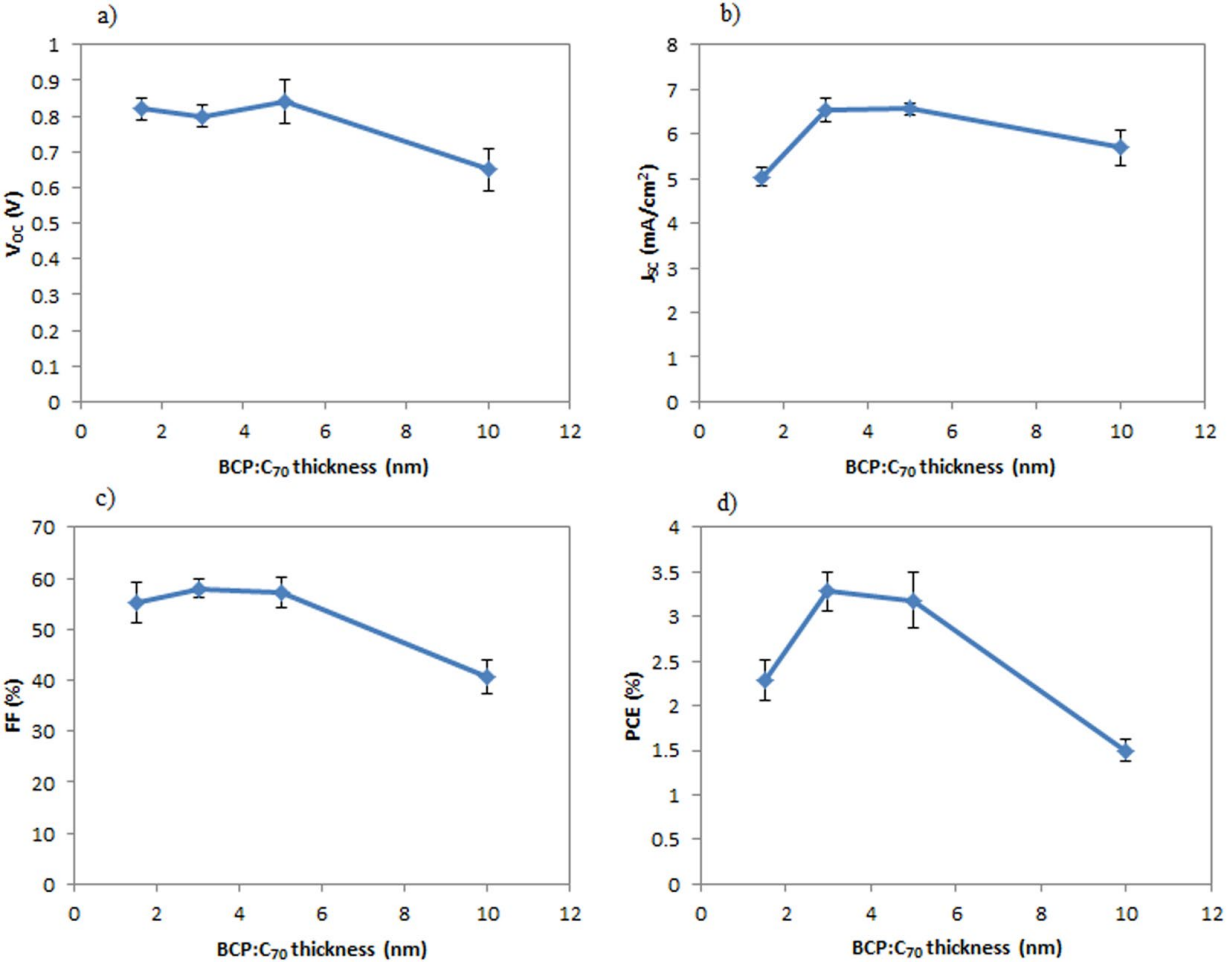
The performance parameters of inverted OPV devices with the various investigated thicknesses of the BCP:C_70_(2:1) ETL and EBL.

As a next step, we turned our attention to optimizing the thickness of the ETL and EBL blend layer in 2 mm^2^ OPV devices. The JV characteristics and performance parameters of the OPV devices are shown in Fig. 3 and in Table 1, respectively. A summary of the performance parameters from Table 1 is plotted in Fig. 4. As shown in Fig. 4b, as the thickness of the BCP:C_70_ layer increases from 1.5 nm to 3 nm, the J_SC_ also increases, which can be well explained by the exciton blocking properties of BCP^46^. When increasing the thickness of the BCP:C_70_ layer above 5 nm, the device performance parameters decrease, and the JV curves show clear s-shape characteristics (Fig. 3). The S-shape could be attributed to charge accumulation close to the active layer and ETL interface^42,47,48^. Charge accumulation close to the thicker ETL and EBL films could take place due to the non-ideal energy level alignment between C_70_ and BCP, although further studies are required to understand that interface in detail. Electron-polaron accumulation at the electron acceptor and blocking interface may lead to exciton-polaron recombination effects^20,31^, a well-known cause for performance drops in OPV devices^49^.

**Table 1:** The performance parameters of the BCP:C_70_ (2:1) OPV devices with the various investigated thicknesses of the BCP:C_70_ ETL and EBL.

BCP:C_70_ thickness (nm)	V_OC_ (V)	J_SC_ (mA/cm_2_)	FF (%)	PCE (%)
1.5	0.82 ± 0.03	5.04 ± 0.22	55.12 ± 3.97	2.28 ± 0.23
**3**	**0.86 **±** 0.03**	**6.54 **±** 0.27**	**57.98 **±** 1.89**	**3.28 **±** 0.22**
5	0.84 ± 0.06	6.56 ± 0.14	57.31 ± 2.96	3.18 ± 0.31
10	0.65 ± 0.06	5.70 ± 0.40	40.58 ± 3.26	1.50 ± 0.12

**Figure 5:**
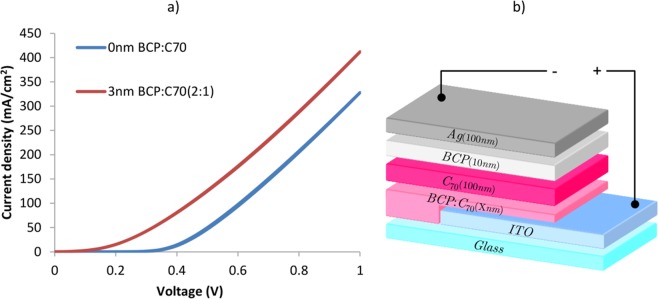
(**a**) The JV characteristics of the electron-only devices (EODs) with 0 and 3 nm BCP:C_70_ (2:1) as ETL. (**b**) The EOD architecture with different thickness of the BCP:C_70_ ETL and EBL.

In order to further elaborate on the electron transport properties of the 3 nm BCP:C_70_ blend ETL and EBL layer, electron-only devices (EODs) were fabricated. The structure of the EODs is shown in Fig. 5b, where 0 and 3 nm of BCP:C_70_ (2:1) blends were investigated. In the EODs, electrons were injected into the devices through the Ag electrode and extracted out at the ITO electrode. The JV characteristics of the EODs with 3 nm BCP:C_70_ ETL show an improvement in the electron extraction properties at the ITO electrode, compared to EODs without the combined ETL and EBL layer (Fig. 5a). Such improvements has also recently been demonstrated for pure, ultrathin BCP layers in small area inverted OPV devices^33^. To this point, the exact energy level alignment scheme across the ITO/BCP:C_70_ interface needs to be examined in detail to point on the origin of the improved electron extraction properties. This highlights the importance of future photoelectron measurements to elucidate the detailed interfacial electronic structure and energetic alignment across the interface.

**Figure 6:**
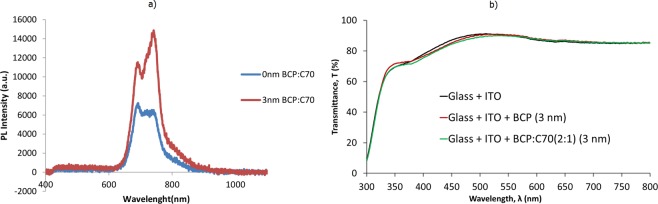
(**a**) Photoluminescence (PL) measurements of C_70_ on ITO with and without the BCP:C_70_ (2:1) ETL and EBLs sandwiched in between. (**b**) Transmittance spectra of ITO-coated glass, ITO-coated glass with 3 nm of BCP and with 3 nmBCP:C_70_ (2:1) layer.

Photoluminescence (PL) intensity measurement was performed in order to elucidate the exciton blocking properties of the BCP:C_70_ blend layers. In Fig. 6a, PL spectra of the pristine C_70_ layer on ITO shows two peaks, a distinct narrow peak at around 690 nm followed by a broader peak at higher wavelengths, corresponding to characteristic electronic and vibrational modes for polycrystalline C_70_, as previously reported^50^. The PL spectra show significant increase in PL intensity from C_70_ when deposited on top of the 3 nm BCP:C_70_ layer, compared to reference stacks based on pure C_70_ layers. The increase in the PL intensity is attributed to the enhanced exciton blocking properties of the BCP:C_70_ blend layers, and thus minimum quenching at the ITO/C_70_ interface^44^. The change in the relative intensity between the two peaks in the PL spectra can be explained by reduced quenching of specific vibronic transitions, upon insertion of the new ETL.

The reduced symmetry of the C_70_ results in more allowed optical transitions and therefore significantly stronger absorption in the visible region compared to symmetrical C_60_^46^. However, for the investigated ETLs, we have used ultra-thin interlayers of BCP:C_70_ (only 3 nm at 2:1 ratio i.e. ~1 nm of C_70_). Hence, the impact of light absorption due to either C_60_ or C_70_ should be negligible in this case. This can be seen in Fig. 6b, where the transmittance spectra of the pure BCP as well as the C_70_ doped BCP:C_70_ (2:1) layer on ITO-coated glass are shown. Clearly 3 nm of BCP or BCP:C_70_ (2:1) show almost no change in optical transmittance, and thus negligible absorption when inserted as an ETL in the inverted device configuration used here. The blended ETL consists of a mixture of C_70_, which efficiently conducts electrons, and the wide energy gap bathocuproine (BCP) that blocks excitons, as demonstrated by our electron only devices and the photoluminescence results. This ETL therefore appears to separate excitons and electrons at the blocking interface as an effective filter, blocking excitons from quenching at the cathode, while promoting electron extraction through the same interlayer in the devices.

**Figure 7:**
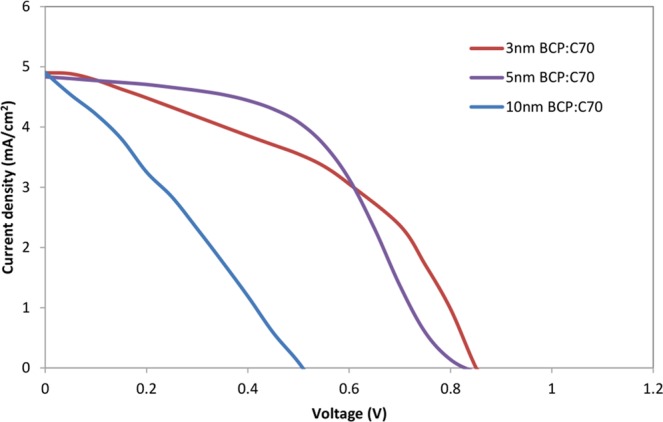
J-V characteristics of large area (100 mm^2^) OPV devices with various thicknesses of the BCP:C_70_ (2:1) ETL and EBLs.

As demonstrated from Fig. 2, the BCP:C_70_ blend layer possesses a smooth surface without BCP aggregation, which otherwise is a main problem in employing BCP in large area inverted devices, due to device shunting^33^. As a final investigation, the optimized BCP:C_70_ blend layers were thus employed in cells with up-scaled device areas of 100 mm^2^, see Fig. 7. As a general observation, a reduced of J_SC_, FF (Table 2) and hence PCE were observed when increasing the active area from 2mm^2^ to 100mm^2^, which in part can be understood from the increased ITO resistance for up-scaled areas^14,33,51^. Devices with 3 nm BCP:C_70_(2:1) show V_OC_ and J_SC_ of 0.85 V and 4.9 mA/cm^2^, respectively, but low FF values of 44% (Table 2). This reduction in FF may be attributed to surface defects of the BCP:C_70_ layer, which may arise due to thickness variations in the very thin ETL and EBL layer. Increasing the BCP:C_70_ thickness leads to an enhancement of the FF, and devices with 5 nm BCP:C_70_(2:1) ETL and EBL show the highest Fill Factor (FF) values of 51%, and power conversion efficiencies (PCE) of 2.04% (Table 2). The performance of the OPV devices reduces significantly when the thickness of the BCP:C_70_ is increased to 10 nm. This can be explained by the increased series resistance and exciton-polaron recombination^20,31^ taking place at the acceptor and blocking layer interface. Initial aggregation could potentially also promote further recombination effects.

**Table 2:** Performance parameters of the large area (100mm^2^) OPV devices with various thicknesses of the BCP:C_70_ (2:1) ETL and EBL.

BCP:C_70_ thickness(nm)	V_OC_ (V)	J_SC_ (mA/cm^2^)	FF (%)	PCE (%)
3 nm	0.85 ± 0.13	4.90 ± 0.29	44.24 ± 1.68	1.84 ± 0.23
**5 nm**	**0.81 **±** 0.07**	**4.84 **±** 0.26**	**51.61 **±** 1.69**	**2.04 **±** 0.17**
10 nm	0.51 ± 0.05	4.90 ± 0.10	28.05 ± 1.06	0.70 ± 0.04

For the large area inverted OPV devices with the BCP:C_70_(2:1) layers, the device yield was at 100%, even for OPV devices with incorporated blends of up to 10 nm in thickness. This is notable when compared to inverted OPV devices based on pure BCP as ETL and EBL, where very low device yields for 100 mm^2^ cells are observed, mainly due to BCP clustering^33^. Doping of BCP with C_70_ thus suppresses the clustering of the BCP molecules, resulting in smoother BCP:C_70_ ETL and EBL on ITO surfaces, giving rise to 100% device yields even for large area devices.

## Conclusion

In this work, development of inverted organic solar cells using mixed bathocuproine:fullerene (BCP:C_70_) electron transport and exciton blocking layers has been demonstrated. Incorporation of C_70_ molecules into the BCP layer suppresses clustering of the BCP molecules, resulting in smooth layers on ITO surfaces, a prerequisite for using them as efficient ETL and EBL in inverted OPV device configurations. While electron-only devices demonstrate improved electron extraction in the cells, photoluminescence studies reveals strong exciton blocking properties of the interface layer. Combining these material properties leads to well performing bilayer C_70_/DBP based inverted devices, reaching power conversion efficiencies up to 3.28%. While BCP clustering is know to be a severe problem for large area OPV cells, leading to significant reduction in device efficiency and device yield, the novel interlayer leads to well-functioning large area cells (100 mm^2^), reaching an impressive device yield of 100%. This work thus demonstrates a viable route for the use of the well-known interlayer material bathocuproine (BCP) in inverted OPV devices.

## Methods

### Materials and Device Fabrication

Pre-patterned ITO coated glass substrates (Kintec Company, Hong Kong) were used for 2 and 100 mm^2^ cell area OPV devices. The sheet resistance of ITO was approximately 15Ω/sq. The substrates were cleaned sequentially in an ultrasonic water bath with detergent, deionized water, Acetone and IPA (10 min for each) then blow dried with a nitrogen gun.

In the first step, OPV devices were fabricated on the cleaned ITO substrates with 2mm^2^ cell areas. The BCP:C_70_ (Sigma-Aldrich, Germany) blend layers with 1.5 nm thickness and different ratio (1:1, 2:1 and 4:1) were grown by co-evaporation, simultaneously depositing from two sublimation sources at a base pressure of 3 × 10^−8^ mbar. This was followed by 30 nm C_70_ at a growth rate of 0.2 Å/s and 20 nm DBP (Luminescence Technology Corp., Taiwan) deposited at 0.3 Å/s without breaking vacuum in between the steps. Then, 10 nm of molybdenum oxide (MoO_3_) (Sigma-Aldrich, Germany) and 100 nm of Silver (Ag) (AESpump ApS, Denmark) were deposited by thermal evaporation at a base pressure of 5 × 10^−7^ mbar. The deposition rates for the MoO_3_ and Ag were 0.3 Å/s and 0.5 Å/s, respectively.

In the second step, 2 mm^2^ OPV devices were fabricated using optimized BCP:C_70_ ratio (2:1) with different thickness (1.5, 3, 5 and 10 nm). The deposition rates for the BCP and C_70_ were 0.2 Å/s and 0.1 Å/s, respectively. Finally, optimized BCP:C_70_ blend layers were used for fabrication of the up-scale OPVs devices (100 mm^2^ cell area). All deposition parameters of the other layers were kept the same as in the first step. Electron-only devices (EODs), having the structure shown in Fig. 5b, were fabricated by sandwiching the BCP:C_70_ mixed layers between the respective contact bottom ITO and top C_70_ (100 nm)/BCP(10 nm)/Ag(100 nm) layers, using the same deposition rates as for OPV device fabrication.

### Device Characterization

All characterizations were performed in an ambient environment. The current density-voltage (J-V) characteristics of the OPV devices were measured using a 2400 source measure unit (Keithley Instruments Inc., USA) and a class AAA solar simulator (Sun 3000, Abet Technologies Inc., USA). The J-V characteristics were measured by applying a voltage sweep from +1 to −0.5 V under a calibrated lamp intensity of 100 mW/cm^2^. Atomic force microscopy (AFM) images were taken using a Veeco Dimension 3100 scanning probe microscope. JV characteristics of the EODs were measured by applying a sweeping voltage from +1 to −1 V using a Keithley 2400 source measure unit (Keithley Instruments Inc., USA). For Photoluminescence (PL) intensity measurements of the ITO/C_70_(100 nm) and ITO/BCP:C_70_(3 nm)/C_70_(100 nm) structures, a microscope objective (Nikon E Plan 50 × 0.75 EPL) with a fluorescence microscope (Nikon Eclipse ME600) connected to a Maya2000Pro Spectrometer (from Ocean optics) was used to record the spectra. A mercury short arc lamp having a filtered excitation wavelength centered between 330–380 nm was used as excitation light source. Transmittance spectra were obtained from a Shimadzu 2700 spectrophotometer.

## Supplementary information

Supplementary Information
