# Supplementary material for: Inverted organic solar cells with non-clustering bathocuproine (BCP) cathode interlayers obtained by fullerene doping
Source: Sci Rep. 2019 Jul 18;9:10422. doi: 10.1038/s41598-019-46854-w (PMC6639309; doi:10.1038/s41598-019-46854-w)
Supplement: Supplementary file 1 — Supplementary Information [file 41598_2019_46854_MOESM1_ESM.docx]

Supporting Information

**Inverted organic solar cells with non-clustering bathocuproine (BCP) cathode interlayers obtained by fullerene doping**

Fatemeh Jafari^1,2^, Bhushan R. Patil^1^, Fatemeh Mohtaram^1,2,3^, André L. Fernandes Cauduro^4^, Horst-Günter Rubahn^1^, Abbas Behjat^2^ and Morten Madsen^1^

^1^ SDU NanoSYD, Mads Clausen Institute, University of Southern Denmark, Alsion 2, 6400 Sønderborg, Denmark

^2^ Atomic and Molecular Groups, Faculty of Physics, Yazd University, Yazd, Iran

^3^ Department of Textile Engineering, Isfahan University of Technology, Isfahan 84156-83111, Iran

^4^ National Center for Electron Microscopy, Molecular Foundry, Lawrence Berkeley National Laboratory, One Cyclotron Road, 94720 Berkeley, California, United States.

Organic solar cells are developed from C_70_ and DBP bilayers as the active layer, using fullerene doped bathocuproine (BCP) as cathode interlayers. Table S1 shows the ratio optimization of the non-clustering fullerene doped BCP layers. Highest performance is achieved at a BCP:C_70_ ratio of 2:1, which was thus further used for thickness optimization (see main manuscript).

Table S1. Performance parameters of the 2mm^2^ OPV devices developed with 1.5 nm BCP:C_70_ ETL and EBL evaporated with different BCP and C_70_ ratios. All the performance data was obtained from average values of in total 18 OPV devices for each ratio, where the error bars represent the standard deviation. Devices based on ‘0nm’ thick layer (last row) are devices made with no ETL layer, i.e., the device stack is: ITO/C70/DBP/MoO_x_/Ag.

| BCP:C_70_ thickness (nm) | BCP:C_70_ ratio | V_OC_ (V) | J_SC_ (mA/cm^2^) | FF (%) | PCE (%) |
| --- | --- | --- | --- | --- | --- |
| 1.5 | 1:1 | 0.73±0.05 | 4.84±0.36 | 50.97±1.73 | 1.81±0.26 |
| 1.5 | **2:1** | **0.82±0.03** | **5.04±0.22** | **55.12±3.97** | **2.28±0.23** |
| 1.5 | 4:1 | 0.77±0.05 | 5.04±0.47 | 48.82±5.10 | 1.89±0.27 |
| 0 | - | 0.85±0.01 | 5.11±0.19 | 52.26±2.89 | 2.27±0.08 |
